# Neuromyelitis optica spectrum disorders: from pathophysiology to therapeutic strategies

**DOI:** 10.1186/s12974-021-02249-1

**Published:** 2021-09-16

**Authors:** Edgar Carnero Contentti, Jorge Correale

**Affiliations:** 1 Neuroimmunology Unit, Department of Neuroscience, Hospital Aleman, Buenos Aires, Argentina; 2 Department of Neurology, Fleni, Buenos Aires, Argentina

**Keywords:** Neuromyelitis optica spectrum disorders (NMOSD), Aquaporin-4-antibody, Astrocyte, Complement, Neuroinflammation, Treatment, Ongoing trials

## Abstract

Neuromyelitis optica (NMO) is a chronic inflammatory autoimmune disease of the central nervous system (CNS) characterized by acute optic neuritis (ON) and transverse myelitis (TM). NMO is caused by a pathogenic serum IgG antibody against the water channel aquoporin 4 (AQP4) in the majority of patients. AQP4-antibody (AQP4-ab) presence is highly specific, and differentiates NMO from multiple sclerosis. It binds to AQP4 channels on astrocytes, triggering activation of the classical complement cascade, causing granulocyte, eosinophil, and lymphocyte infiltration, culminating in injury first to astrocyte, then oligodendrocytes followed by demyelination and neuronal loss. NMO spectrum disorder (NMOSD) has recently been defined and stratified based on AQP4-ab serology status. Most NMOSD patients experience severe relapses leading to permanent neurologic disability, making suppression of relapse frequency and severity, the primary objective in disease management. The most common treatments used for relapses are steroids and plasma exchange.

Currently, long-term NMOSD relapse prevention includes off-label use of immunosuppressants, particularly rituximab. In the last 2 years however, three pivotal clinical trials have expanded the spectrum of drugs available for NMOSD patients. Phase III studies have shown significant relapse reduction compared to placebo in AQP4-ab-positive patients treated with satralizumab, an interleukin-6 receptor (IL-6R) inhibitor, inebilizumab, an antibody against CD19^+^ B cells; and eculizumab, an antibody blocking the C5 component of complement. In light of the new evidence on NMOSD pathophysiology and of preliminary results from ongoing trials with new drugs, we present this descriptive review, highlighting promising treatment modalities as well as auspicious preclinical and clinical studies.

## Background

**Fig. 1:**
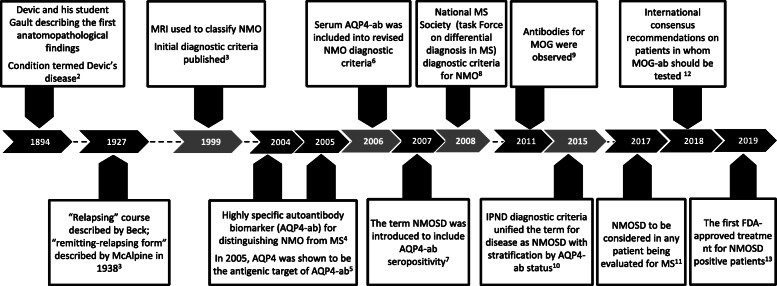
Timeline and relevant milestones in NMOSD. During the last two decades, significant advances have been made in NMOSD, including: introduction of new diagnostic criteria (gray arrows), identification of biomarkers, better characterization of clinical phenotypes, improved prognosis and new therapeutic approaches (black arrows). *AQP4* aquaporin-4, *AQP4*-*ab* aquaporin-4-antibodies, *IgG* immunoglobulin G, *IPND* International Panel for NMO Diagnosis, *MOG* myelin-oligodendrocyte glycoprotein, *NMO* neuromyelitis optica, *NMOSD* neuromyelitis optica spectrum disorder, *TM* transverse myelitis

Neuromyelitis optica (NMO) is a chronic inflammatory autoimmune disease of the central nervous system (CNS) associated with a characteristic pattern of astrocyte dysfunction and loss, resulting in secondary demyelination and neurodegeneration [1]. Originally known as Devic’s disease, NMO mostly follows a relapsing course, and was long considered a severe variant of multiple sclerosis (MS). For over 100 years, very little was known on the pathogenesis of the disease, and evidence-based treatments were scarce (Fig. 1) [2–13].

In 2004, discovery of a pathogenic NMO-associated IgG antibody, targeting the water channel membrane protein aquaporin-4 (AQP4), was an important milestone in differentiating NMO from MS [4]. After varying forms of clinical presentation were described for the disease, the term NMO spectrum disorder (NMOSD) was introduced in 2007 [7]. AQP4 is highly concentrated on astrocyte end-feet in the CNS. Although pathogenic AQP4-antibodies (AQP4-ab) are found exclusively in patients with NMO [5], approximately 20–30% of NMOSD patients are seronegative for AQP4-ab. Up to 42% of these AQP4-ab-negative NMOSD patients have IgG antibodies against myelin oligodendrocyte glycoprotein (MOG-ab) [9, 12, 14], increasingly recognized as defining an overlapping clinical syndrome, also meeting a clinical diagnosis of NMOSD [15, 16]. Binding of AQP4-ab to astrocyte AQP4 channels triggers classical complement cascade activation, followed by granulocyte, eosinophil, and lymphocyte infiltration, culminating in injury first to astrocytes, then oligodendrocytes, demyelination, neuronal loss, and neurodegeneration [1].

Most recently, NMOSD was defined and stratified based on AQP4-ab serology status [10]. Additionally, six core clinical characteristics were described, and brain and spinal cord magnetic resonance (MRI) findings suggestive of NMOSD were better defined [10]. Optic neuritis ([ON]; often severe, may be bilateral), transverse myelitis ([TM]; often complete and may be accompanied by paroxysmal tonic spasms, pruritus or pain), and area postrema syndrome ([APS]; intractable hiccups or nausea and vomiting) are the cardinal symptoms of NMOSD, although some patients can also have brain or brainstem involvement (i.e., brainstem syndrome, acute diencephalic syndrome and symptomatic cerebral syndrome), which can manifest with a variety of different symptoms [10]. In AQP4-ab-negative NMOSD patients, the role of MOG-ab still require further clarification [15, 16].

Although NMOSD and MOGAD are two antibody-mediated entities, it is clear that both have different targets [9, 12, 14]. The frequency of MOG-ab and AQP4-ab coexistence was exceptionally reported [9, 15, 16], suggesting that both have different immunopathogenic mechanisms. AQP4-ab-positive NMOSD is characterized by AQP4 loss, dystrophic astrocytes, and absence of cortical demyelination [14–16]. By contrast, MOGAD pathology is characterized by the coexistence of perivenous and confluent primary demyelination with partial axonal preservation and reactive gliosis in the white and gray matter, with particular abundance of intracortical demyelinating lesions [14]. This occurs on the background of CD4-dominated T cells and granulocytic inflammatory infiltrates.

Contrary to classical AQP4-ab-positive NMOSD, in MOGAD the expression of AQP4 is preserved [14]. These findings, added to the clinical and radiological differences, clearly demonstrate that AQP4-ab-positive NMOSD and MOGAD are two different entities [14–16].

NMOSD is frequently associated with antibody-mediated autoimmune disorders including myasthenia gravis, lupus, Sjogren syndrome, among others [17, 18]. Myasthenia gravis coexists more frequently than expected, with NMOSD usually occurring several years after myasthenia diagnosis [7, 18].

Immunotherapy usually used to treat MS patients, including drugs such as interferon beta, fingolimod, natalizumab, and alemtuzumab, is ineffective in NMOSD patients and may even increase annualized relapse rates (ARR) [17]. Currently, NMOSD treatment is divided into treatment of acute episodes, individual symptom management, and long-term relapse prevention.

Long-term relapse prevention includes treatments based on data from retrospective observations, and prospective observational studies without control groups. The most commonly used treatments include azathioprine (AZA), mycophenolate (MMF), and rituximab [18]. In the last 2 years however, four pivotal randomized clinical trials (RCT) have expanded the spectrum of drugs available for NMOSD patients. Phase 3 studies have shown significant relapse reduction compared to placebo, in patients treated with monoclonal antibodies against the interleukin-6 receptor ([IL-6R]; satralizumab) [19, 20], against CD 19 present on CD19-expressing B cells (inebilizumab) [21], and against the C5 fraction of complement (eculizumab) [13]. This effect was particularly found in AQP4-ab-positive NMOSD patients in all three trials [13, 19, 20].

We therefore present a review on the most relevant findings as well as the auspicious preclinical and clinical study results, in light of additional evidence on molecular mechanisms underlying NMOSD, and ongoing trials of new drugs for treating this condition.

## Pathophysiology of neuromyelitis optica spectrum disorders

### NMOSD is an AQP4-ab-associated disease

The discovery of selective AQP4-ab binding to AQP4 changed our understanding of NMOSD pathogenesis. What had been considered a primarily demyelinating disease is now categorized as an autoimmune astrocytopathy [1, 5]. AQP4 is a bi-directional, osmosis-driven water channel, found at highest concentration in perivascular and peripheral astrocyte endfeet, as well as in ependymal cell membranes [22]. In humans, AQP4 monomers are expressed in astrocytes in two isoforms: M1-AQP4 and M23-AQP4. Both isoforms have identical extracellular domain residues, but M1-AQP4 has 22 more amino acids at the cytoplasmic N terminus. However, AQP4-ab binding to the ectodomain of astrocytic AQP4 has isoform-specific outcomes. M1-AQP4 is completely internalized, whereas M23-AQP4 resists internalization and is aggregated into larger-order orthogonal arrays of particles (OAPs) [23], a process facilitated by M1-AQP4 deficiency. OAP function under physiological conditions is still unknown. However, alteration in OAP assemblies have been reported in several CNS diseases, and are required for NMO-IgG to recognize conformational AQP4 epitopes [24]. OAPs may also be critical for binding of the complement component C1q, to clustered AQP4-ab [25, 26].

Neuropathological, clinical, and animal studies provide evidence of a role of AQP4-ab, in NMOSD pathogenesis. CNS lesions in NMOSD patients are characterized by IgG, IgM, and complement deposits with a rosette pattern, most prominent around vessels, as well as cellular infiltrates of granulocytes (neutrophils and eosinophils) macrophages/microglia and T cells [27]. One key feature is AQP4 loss on astrocytes. In certain lesions however, other typical astrocytic markers, such as glial fibrillary acidic protein (GFAP) and S-100β, are still detectable, indicating AQP4 loss precedes astrocyte death [27, 28]. Ultimately, preservation or secondary loss of neurons and associated demyelination will depend on disease severity. Demyelination affects both gray and white matter, sometimes with necrosis and cavitation, and thickened, hyalinized vessels [27, 29]. These findings suggest the autoimmune response in NMOSD primarily affects astrocytes and is initiated by autoantibody-mediated loss of AQP4.

Clinical observations also support the hypothesis that AQP4-ab cause NMOSD and are highly specific [7, 25]. They can be detected in sera of most patients [25, 30], and levels of both AQP4-ab and AQP4-ab–producing plasmablasts correlate with disease activity [25, 30–32]. In addition, AQP4-ab presence may predict future relapses [33, 34]. Although AQP4-ab thresholds triggering clinical relapses have not been established, and serum levels show wide variations both in individual patients and between patients, lesion distribution tends to correlate with areas of highest AQP4 expression and blood-brain barrier (BBB) permeability (e.g., area postrema) [7, 35, 36]. Another indirect sign pointing to the role of AQP4-ab-related disease mechanisms comes from the success of therapeutic strategies targeting humoral immune responses, such as plasma exchange [37, 38], or use of B-cell depleting therapies like rituximab [39, 40].

Further evidence has been observed in animal experiments. No animal models replicating spontaneous AQP4 autoimmunity exist. However, passive intravenous [41] or intraperitoneal transfer [42] of purified AQP4-ab from NMOSD patients and of human complement to rodents causes early AQP4 loss and astrocyte cytotoxicity, identical pathological features to those found in NMOSD [41, 42].

Growing evidence indicates AQP4-ab are synthetized peripherally, rather than intrathecally, subsequently entering the CNS through a disrupted BBB [25, 33]. Few NMOSD cases show AQP4-ab exclusively in CSF [43]. In line with this assumption AQP4-ab-plasmablasts are selectively increased in NMOSD patients, and maintained by elevated levels of IL-6 [44]. It has been postulated that AQP4-ab require an inflammatory background and BBB disruption, to induce CNS lesions [45]. Indeed, relative high levels of AQP4-ab are detected in many patients, even during remission [31]. Other evidence has indicated an inflammatory environment may not always be necessary for AQP4-ab to cross the BBB [46], as they may enter the CNS via circumventricular organs with fenestrated capillaries, or through blood vessels in the meninges or parenchyma [47]. AQP4-ab itself could induce direct damage to the BBB, since astrocytic endfeet is a constitutive element of the BBB [42].

### The role of B cells and plasma cells, in NMOSD

The pathogenic role of AQP4-ab highlights the importance of B cells and plasma cells in NMOSD [48, 49]. CD19^int^CD27^high^CD38^high^CD180^−^ B cells are selectively expanded in peripheral blood of patients. These cells have the phenotypic features of plasmablasts, and secrete AQP4-ab following IL-6 stimulation [50]. Notably, B cells found in CSF of NMOSD patients display signals of somatic B cell hypermutation, indicative of antigen recognition within the CNS. Similarly, elevated levels of B cell activating factor (BAFF), proliferation-inducing ligand (APRIL), CXCL13, and IL-6 are all found in CSF from NMOSD patients, likely exerting a critical role in AQP4-ab-producing cell recruitment and maintenance [51, 52]. Interestingly, eosinophils infiltrating the CNS (see below) may facilitate plasma cell survival and AQP4-ab production, through production of APRIL, IL-6, and IL-5 [48]. B cell contribution to NMOSD pathogenesis, however, may extend beyond the production of AQP4-ab. B cells could also play a critical role as antigen presenting cells for development of follicular effector T cells, which participate in B cell differentiation and isotype switching [53, 54], closing a positive feedback loop of potentially pathogenic B and T cell interaction. Furthermore, bystander activation may also result in production of B cell cytokines promoting NMOSD activity, including IL-6, TNF-α, and GM-CSF. IL-6 secretion by pro-inflammatory memory B cells in NMOSD may increase disease activity through different mechanisms, namely by (i) promoting plasmablast survival, (ii) stimulating AQP4-ab production, (iii) disrupting BBB integrity, and (iv) promoting pathogenic Th17 cell differentiation [55]. Lastly, a decrease in regulatory B cell numbers may impair function or reduce levels of IL-10, also worsening disease [56], although this mechanism needs further study.

### T cells and NMOSD

AQP4-ab in NMO serum are IgG1, a subclass of mature IgG that requires help from T cells, indicating that AQP4-specific CD4^+^ T cells participate in the genesis of this adaptive humoral response [57]. Paucity of T cells in NMOSD lesions probably indicates they are not directly involved in lesion formation [25]. However, several observations show that T cells may act in the periphery disrupting tolerance and contributing to AQP4-ab production [25, 58]. Peripheral blood T cells from NMO patients and healthy controls proliferate in response to intact AQP4 and AQP4 peptides, with a robust T cell response in NMOSD patients to p61–80 [57]. Interestingly, AQP4 63–76 peptide contains the predicted binding motif for HLA-DRB1*0301 and HLA-DRB3*0202, two haplotypes overrepresented in some NMOSD populations [59, 60]. Several studies have shown that AQP4 antigenic stimulation polarizes the immune response toward a Th17 repertoire, and to secretion of Th17-associated cytokines such as IL-6 and IL-21 [61]. Th17 cells may compromise BBB integrity via IL-17 secretion, promoting endothelial activation, and stimulating trans-endothelial migration of neutrophils [62]. Meanwhile, increased CSF levels of IL-6 in NMOSD patients may favor survival of AQP4-specific Th17 cells, and inhibit FOXP3^+^ regulatory T cells at the same time [63]. Collectively, these observations highlight the potential role of AQP4-specific T cells as drivers of adaptive humoral as well as cellular immune responses in NMO pathogenesis.

### Innate immunity in NMOSD

AQP4-ab binds to extracellular epitopes of AQP4 present on the astrocyte plasma membrane. This triggers astrocyte injury through complement-dependent cytotoxicity (CDC) and antibody-dependent cellular cytotoxicity (ADCC). In CDC, classical pathway activation begins when the multivalent protein C1q, binds to the conformational Fc determinant on IgG or IgM antibody–antigen complexes, producing cellular injury by formation of a pore-like membrane attack complex (MAC) [64]. In addition to MAC formation, complement activation produces factors C3a and C5a, which increase vascular permeability and provide a chemotactic gradient, resulting in recruitment of immune effector cells through the BBB, including neutrophils, basophils, eosinophils, mast cells, and macrophages [65]. Increased numbers of both neutrophils and eosinophils are found in the CSF of NMOSD patients [66], as well as in autopsy cases of NMO [29]. In ADCC, binding of neutrophils, macrophages, and NK cells to the Fc region of AQP4-ab, through Fcγ receptors, causes activation and degranulation, resulting in NMOSD lesions [67]. Both mechanisms may also cause cytotoxicity to nearby cells including oligodendrocytes and neurons, through bystander mechanisms [68, 69]. The role of complement in NMOSD pathogenesis is highlighted by the demonstration of pronounced perivascular deposition of immunoglobulins, mainly IgM, and complement C9neo antigen (the residual component of MAC), in active demyelinating lesions associated with prominent vascular fibrosis and hyalinization, both in active and inactive lesions [29].

Without complement, astrocytic membranes remain intact, but AQP4 is endocytosed with concomitant loss of Na^+^-dependent glutamate transport and loss of the excitatory amino acid transporter 2 (EAAT2), suggesting that EAAT2 and AQP4 exist in astrocytic membranes as a macromolecular complex. Impairment in glutamatergic homeostasis may contribute to neurotoxic events that lead to neuronal death, oligodendrocyte dysfunction, and consequent demyelination [70].

In addition to decrease in AQP4, loss of astrocytes, neuronal injury, and demyelination, microglia activation and macrophage infiltration are also prominent in NMOSD. Microglia/macrophage distribution is confined to regions with high expression of AQP4, surrounded by signs of complement activation [71]. The potential role of microglia activation in NMOSD has been recently studied in an animal model. AQP4-ab is known to elicit significant production of complement fraction C3 by astrocytes [72], and microglia express C3a receptor. Therefore, astrocytes can promote microglia activation through C3a, particularly during the pre-cytolytic phase [73]. Microglia activation also induces production of complement C1q, which in turn can promote axonal damage and neurodegeneration, independent of complement [74, 75].

Studies in experimental animal models as well as in human NMOSD lesions show polymorphonuclear leukocytes (PMNs) as the key determinants of BBB permeability and NMOSD lesion formation. Indeed, depletion of blood PMNs significantly reduces BBB disruption, a finding that has been confirmed in vivo using marked albumin tracer [76].

CSF neutrophil counts are elevated in about 60% of untreated NMOSD patients during relapses, but in only 20% during remission [66]. One study showed NMO patient sera contained elevated levels of neutrophil chemo-attractants CXCL5 and CXCL8, and the neutrophil protease, elastase [77]. In an NMO mouse model, tissue damage was dampened by neutrophil depletion and enhanced by neutrophil increase [78]. Immunostaining for neutrophil elastase (NE) showed perivascular neutrophils were degranulated, suggesting those circulating and entering the CNS, participated in NMOSD lesion development, through NE–dependent mechanisms. Furthermore, sivelestat, a small-molecule NE inhibitor, reduced disease severity [78].

One striking feature of active NMO lesions in the spinal cord is intense perivascular and meningeal infiltration of eosinophils, as well as of CCR3, the principal receptor for the chemokine eotaxin, and a potent eosinophil chemo-attractant [29]. Once activated, eosinophils release several cytotoxic proteins including eosinophil cationic protein (ECP), eosinophil-derived neurotoxin (EDN), eosinophil peroxidase (EPX), and major basic protein (MBP) [79].

**Fig. 2:**
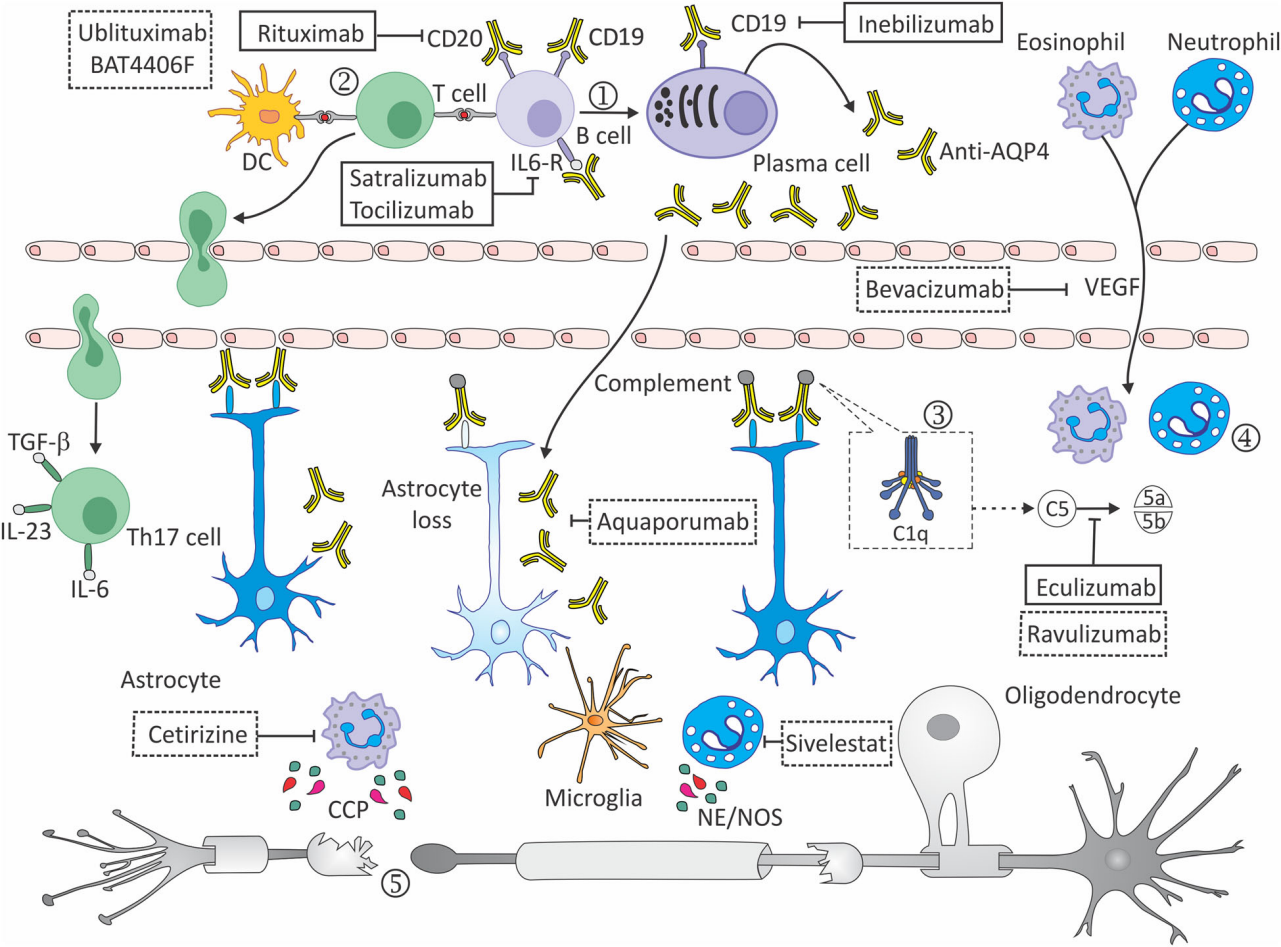
Pathophysiologic mechanisms and therapeutic targets for approved and experimental treatment options in NMOSD. AQP4-specific B cells differentiate in the periphery to plasma cells capable of producing anti-AQP4 antibodies (1), which penetrate the CNS and are deposited mainly on the feet of astrocytes. Specific T cells interact with B cells or dendritic cells, and in the presence of IL-6, IL-23, and TGF-β differentiate into Th17 cells. These in turn penetrate the CNS, facilitate the passage of AQP4-ab into the CNS via opening the blood brain barrier (BBB), and contribute to the recruitment of neutrophils (2). This inflammatory environment activates complement through C1q which binds to anti-AQP4-ab, induces C5 cleavage into activated fractions C5a and C5b, causing astrocyte injury through complement-dependent cytotoxicity (CDC) and antibody-dependent cellular cytotoxicity (ADCC). When C1q binds to conformational Fc determinants on IgG or IgM antibody–antigen complexes, it produces cellular injury by formation of the pore-like membrane attack complex (MAC) (3). In addition to MAC formation, complement activation produces factors C3a and C5a, which together with VEGF increase vascular permeability and provide a chemotactic gradient, resulting in recruitment of neutrophils, eosinophils, basophils, mast cells, NK cells and macrophages (4). These cells produce complement-independent damage of astrocytes through ADCC or degranulation involving Fc receptors. Mechanisms described above may also generate cytotoxicity in neighboring cells including oligodendrocytes and neurons through bystander effects (5). Experimental treatments or those in ongoing studies are represented in dotted line spaces. *AQP4* aquaporin-4, *CCP* cytotoxic cationic proteins, *IL* interleukin, *NE* neutrophil elastase, *NOS* nitric oxide species, *VEGF* vascular endothelial growth factor

Cerebrospinal fluid from patients with NMO contains higher levels of eotaxin-2, eotaxin-3, and ECP compared to healthy controls or multiple sclerosis patients [52]. In addition, stimulation of CSF cells from NMO patients with MOG results in increased IL-5 production [52]. Taken together, these observations indicate eosinophils can cause neural tissue damage in NMO, through ADCC and degranulation. The main pathological mechanisms involved in NMOSD are summarized in Fig. 2.

## Treatment of neuromyelitis optica spectrum disorders

For decades, NMOSD treatment has been based on retrospective case series and consensus guidelines. Better knowledge of underlying disease mechanisms has allowed the identification of new therapeutic targets. Results from RCT targeting different pathways have been recently published, providing Class I evidence for use of different monoclonal antibodies [13, 19–21, 40, 80]. Additional therapies in development or undergoing trials for NMOSD have arisen as a result of improvements in our understanding of the pathogenesis of the disease.

### Past and present treatments for acute disease phases

Acute treatment is critically important in NMOSD as exacerbations result in severe residual disability. Therefore, relapse therapies should be started early and aggressively [18]. Acute treatment objectives include suppressing acute inflammatory attacks, restricting CNS damage, and improving long-term neurological function.

Relapses are commonly treated with high-dose IV methylprednisolone (IVMP) 1 g/daily for 3–7 days, followed by tapered oral steroids [81]. Complete recovery after relapse has been observed in up to 35% of patients treated with IVMP [82, 83]. Timing is critical, IVMP within 5 days of AQP4 ON onset increases complete visual acuity (VA) recovery compared to starting later [84].

Plasma exchange (PLEX) every other day for 2 weeks (1.5 L volume, 5–7 treatments) or immunoadsorption are recommended within 5 days from NMOSD relapse onset, when response to IVMP is poor or absent [18]. PLEX can also be administered as first line therapy or simultaneously with IVMP in severe cases. In serious TM relapses, early PLEX was linked to full recovery compared to high-dose steroids (OR = 4.38, *p* = 0.006) [85]. Similarly, time from relapse onset to start of PLEX was a robust predictor of complete remission (40% within 2 days of symptom onset vs. 3.2% after 6 days) [84]. Moreover, 51% of patients treated with IVMP for 5 days followed by PLEX, recovered pre-relapse baseline status, compared with 16.6% of patients treated only with IVMP [82]. Degree of recovery decreased from 50% when PLEX was given immediately, to 1–5% when started after day 20, emphasizing the importance of early treatment [86]. If response is poor, IV immunoglobulin-G therapy (IVIgG) can be used. In a retrospective study on 10 NMOSD patients unresponsive to IVMP, IVIgG was effective in 50% of patients [87]. Unfortunately, a multicenter, single-blind, parallel-group, RCT on IVIG compared to standard therapy for TM treatment in adults and children (STRIVE study [NCT02398994]) was discontinued due to difficulties recruiting patients [88]. Recently, a retrospective study reported high-dose IVMP plus IVIgG was superior to high-dose of IVMP alone [89]. Further studies will be needed to confirm these findings.

### Future treatments for acute disease

New therapies for acute NMOSD relapses, including bevacizumab, ublituximab, NPB-1, and HBM9161, are currently under investigation. Mechanism of action, doses used, and RCT results observed with these treatments are summarized in Tables 1 and 2.

**Table 1:** Mechanism of action of on-label and off-label therapies, and drugs in clinical trials used in the treatment of NMOSD

Acute treatment: future era	
Drug/Dose/Route of administration	
**Bevacizumab** [90] intravenous infusion 10 mg/kg intravenous infusion at onset of exacerbation and, if needed, a second time during the plasma exchange phase	Bevacizumab directly binds vascular endothelial growth factor (VEGF) to inhibit angiogenesis
**Ublituximab** [91, 92] Intravenous 450 mg once on day 1, plus steroids 1000 mg intravenously daily on days 1–5	Ublituximab is a monoclonal antibody that specifically binds to the trans-membrane antigen CD20. Binding induces an immune response that causes lysis of B cells.
**NPB-01** (NCT01845584) Intravenous immunoglobulin 400 mg/kg/day for five consecutive days	IgG can inactivate auto-reactive T-cells by competing for, and interrupting their interaction with, antigen presenting cells [87, 88].
**HBM 9161** (NCT04227470) injection, 340 mg or 680 mg weekly administered subcutaneously for a period of 4 weeks.	HBM9161(HL161BKN) is a human monoclonal antibody. HBM9161 targets FcRn by blocking the FcRn IgG-Fc binding site and accelerating the degradation of IgG, reducing total IgG level in blood (including pathological IgG). The serum AQP4-IgG associated with NMOSD is a pathological IgG, so the combination of standard of care which is intravenous methylprednisolone with HBM9161 is expected to rapidly reduce AQP4-IgG levels.
**Long-term relapse prevention treatment: old era**	
**Azathioprine (AZA)** [93–96] Oral Target dose: 2–3 mg/kg/daily in divided doses	Purine analog that converts to 6-mercaptopurine, its active metabolite, and thioguanine due to the action of hypoxanthine-guanine phosphoribosyl transferase and thiopurine methyltransferase enzymes. Inhibits purine synthesis resulting in the inhibition of DNA, RNA, and protein synthesis. AZA is absorbed rapidly through the GI system and does not penetrate the blood-brain barrier.
**Mycofenolate mofetil (MMF)** [97–99] Oral Target dose: 750–1500 mg twice a day (median dose: 1 g twice a day)	Prodrug of mycophenolic acid, an inhibitor of inosine-5'-monophosphate dehydrogenase (antimetabolite), which is the first of two enzymes involved in the conversion of inosine monophosphate (IMP) to guanosine monophosphate (GMP). It is normally converted to GDP, GTP, and dGTP. Mycophenolic acid treatment decreases guanine nucleotide pools in lymphocytes.
**Rituximab (RTX) [**100–106] Intravenous Induction: 1 g with re-treatment at 2 weeks or 375 mg/m^2^ body surface area once weekly for 4 weeks. Maintenance: 1 g with retreatment at 2 weeks every 6 mo. or one infusion of 375 mg/m^2^ every 6 mo.	Chimeric monoclonal antibody (IgG1) against human CD20. Its binds to CD20, a protein expressed primarily on B cells (pre-B, naïve and memory B cells), reducing B cell activity (elimination of autoreactive B cell) through subsequent cytotoxic mechanisms, inducing regulatory B cells.
**Tocilizumab (TCZ)[**80, 107–109] Intravenous 8 mg/kg every 4 weeks	Humanized monoclonal antibody (IgG1) genetically engineered from mouse antihuman anti-interleukin 6 receptor (IL-6R) antibody. It recognizes the IL-6 binding site of the human IL-6R and inhibits IL-6 signaling through competitive blockade of the IL-6 binding site (membrane-bound and soluble IL-6 receptors)
**Long-term relapse prevention treatment: present Era**	
**Eculizumab (ECZ)** [13] Intravenous 900 mg weekly during the first four doses starting on day 1, followed by 1200 mg every 2 weeks starting at week 4.	Humanized monoclonal antibody (IgG2/IgG4) inhibiting terminal complement protein C5 by preventing cleavage from C5 to activated fractions C5a (pro-inflammatory peptide involved in chemotaxis, cytokine release and vasodilation) and C5b (a membrane constituent which attacks complex C5b-9).
**Satralizumab** [19, 20] Subcutaneous 120 mg at weeks 0, 2, and 4 and then every 4 weeks	Humanized IL-6R monoclonal antibody type IgG2 (recycling technology). Binds to membrane IL-6R and is internalized in the endosome. It can dissociate IL-6R under acidic conditions in lysosomes and be recycled to the plasma via the neonatal Fc receptor (FcRn) instead of being degraded in lysosomes.
**Inebilizumab** [21**]** Intravenous 300 mg in 2 doses on open-label days 1 and 15 and then 300 mg every 6 mo.	Humanized monoclonal antibody (IgG1) against CD19 (pro-B, pre-B, naïve and memory B cells), which produces rapid depletion of circulating B cells, including autoantibody-secreting plasmablasts and CD19-expressing plasma cells. CD19 is exclusively expressed on B cells.
**Long-term relapse prevention treatment: future era**	
**Telitacicept** Subcutaneous 160 mg weekly	Recombinant transmembrane activator and calcium modulator and cyclophilin ligand interactor (TACI-Fc; located on CD27+ memory B cells and plasma cells) fusion antibody that works by binding to two cell-signaling molecules, B lymphocyte stimulator (BLyS), and a proliferation-inducing ligand (APRIL), both are a member of the tumor necrosis factor (TNF) family [115].
**Ravulizumab** Intravenous Infusion on day 1, followed by weight-based maintenance doses on day 15, then once every 8 weeks	Second-generation anti-C5 monoclonal antibody (binds to complement protein 5 (C5) and blocks its activation by complement pathway convertase, thus inhibiting C5 cleavage into fragments C5a and C5b, engineered from eculizumab. It is a long-lasting recycling IgG monoclonal antibody with increased affinity for FcRn and rapid endosomal dissociation of the ravulizumab-C5 complex, allowing lysosomal degradation of C5 while recycling ravulizumab to the vascular space through the FcRn [113].
**Bortezomib[**114] Subcutaneous 1 mg/m^2^ of body surface area on days 1, 4, 8, and 11 per cycle followed by a 10-day treatment-free interval.	Binds the catalytic site of the 26S proteasome with high affinity and specificity leading to elimination of both plasmablasts and plasma cells by activation of the unfolded terminal protein response. Bortezomib may protect astrocytes from NFκB-dependent inflammatory damage in early events in NMOSD pathogenesis.
**Cetirizine (add-on)** [115] **Oral** 10 mg each day	Cetirizine (antihistaminic) could prevent damage by blocking eosinophils which have been implicated in the pathophysiology of NMOSD.
**BAT4406F** Intravenous Open-label dose escalation starting from 20 mg.	Fully humanized anti-CD20 monoclonal antibody
**SHR1459** Oral Tablets taken once daily	Bruton’s tyrosine kinase (BTK) inhibitor. BTK plays a crucial role in B cell development by transmitting intracellular signals from the pre-B cell receptor
**Precinical study**	
**Aquaporumab (mAb-53) (animal model)[**119] mAb-53 has not been clinically applied to patients	Aquaporumab is an engineered monoclonal antibody with high affinity for AQP4 channels that contain Fc mutations blocking cell- and complement-mediated cytotoxicity effector functions (possible mechanism of competitive inhibition as a steric inhibitor). Aquaporumab has shown beneficial effects in an NMOSD mouse model, but has not been clinically tested in NMOSD patients.

**Table 2:** Therapeutic options for NMOSD-related relapses

Drug	Study design	Study phase / ClinicalTrials.gov Identifier(status 01/2021)	Number of patients (randomization)	NMOSD serostatus	Follow-up	Disability (EDSS stabilization or improvement)	Safety concerns
**Bevacizumab**	Single-center, Open Label Trial (USA)	Phase 1 add-on therapy (completed) NCT01777412	10	AQP4-ab + (*n* = 6) and – (*n* = 4)	91 days after admission	at baseline: 3.5 (2–7) at FU: 3 (1.75–6.5)	UTI that required hospitalization and improved with specific Tx
**Ublituximab**	Single-center, Open Label Trial (USA)	Phase 1 add-on therapy (completed) NCT02276963	6 (5 completed the study)	AQP4-ab +	90 days after admission	at baseline: 6.5 (5.25-7.5) at FU (*n* = 3): 4 (2–8)	Leukopenia (*n* = 1) headache and body ache (*n* = 3)
**NPB-01**	Single-center, Open Label Trial (Japan)	Phase 2 add-on therapy (completed) NCT01845584	7	AQP4-ab +	Time frame: 29 days	NA	NA
**HBM 9161**	Non-randomized, open label, dose exploration study (China)	Phase 3 study (Active, recruiting) NCT04227470	12 (estimated enrollment)	AQP4-ab +	Time frame: 189 days	NA	NA
**Immunoadsorption or Plasma Exchange**	Prospective, Multicenter, Single-blind, Randomized study (China)	Phase 2 study (not yet recruiting) NCT04064944	144 (estimated enrollment)	AQP4-ab +	Time frame: 4 weeks after the last treatment	NA	NA

*FU* follow-up, *NA* not available, *USA* United States of America, *AQP4*-*ab* + aquaporin-4 antibodies positive, *NMOSD* neuromyelitis optica spectrum disorders, *EDSS* Expanded disability Status Scale, *UTI* urinary tract infection

IV bevacizumab was evaluated in a phase 1b trial, as add-on therapy for treatment of ON and/or TM in NMOSD. Bevacizumab, an anti-angiogenic compound, which can restore the BBB proved effective and safe in 10 patients, with none requiring escalation to PLEX after high-dose IVMP plus IV bevacizumab, suggesting that this is a potent combination [90]. Ublituximab (LFB-R603) is a monoclonal antibody specifically binding to the trans-membrane antigen CD20, shortening infusion duration and lowering doses compared to other anti-CD20 monoclonal antibodies [91]. In a phase 1 open-label study to assess safety of acute B cell depletion in NMOSD patients with ON or TM relapses, patients received IV ublituximab at relapse onset plus high-dose of IVMP for 5 days. Ublituximab proved to be safe in all 5 patients and 4 of 5 showed improved disability scores [92]. Subcutaneous injection of HBM9161 is being evaluated in a phase 1, open-label dose exploration study, investigating safety/tolerability, pharmacodynamics, and efficacy in NMOSD patients experiencing relapses (NCT04227470). A phase 2 RCT of IVIgG (NPB-01) in AQP4-ab-positive NMOSD patients did not improve response when added to IVMP, but detailed results are not available. Finally, a new study comparing efficacy and safety of immunoadsorption and PLEX for acute relapse of refractory NMOSD (CAMPUS; NCT04064944) has been announced, but is not recruiting yet.

### Long-term relapse prevention treatment: overall principles and objectives

To minimize permanent neurologic disability, long-term relapse prevention treatment is recommended for all patients who are diagnosed with NMOSD [18]. So far, no standard management has been agreed upon for first-line treatment or treatment switching. Figure 2 summarizes the main therapeutic targets in NMOSD.

Off label use of some older immunosuppressive agents such as AZA [93–96], MMF [97–99], and rituximab [100–106] have shown reductions in ARR, with disability stabilization in retrospective, prospective, and meta-analysis studies. Rituximab was also recently evaluated in a RCT (RIN-1) [40]. Tocilizumab has shown promising reductions in NMOSD relapse activity [80]. Advances in the understanding of immune mechanisms involved in NMOSD have led to three recent RCTs evaluating targeted monoclonal antibodies [13, 19–21, 40, 80]. Eculizumab was the first monoclonal approved by the Food and Drug Administration to prevent NMOSD relapses in AQP4-ab-positive patients in June 2019 [13], followed by Inebilizumab (June 2020) [21] and satralizumab (August 2020) [19, 20]. Primary outcome in all three trials was efficacy and safety in delaying first relapse after treatment [13, 19–21, 40, 80]. Mechanism of action, doses, and most relevant results of these drugs are summarized in Tables 1 and 3.

**Table 3:** Therapeutic options for long-term relapse prevention in NMOSD

Drug	Study design	Study phase/ClinicalTrials.gov Identifier (status 01/2021)	Number of patients (randomization)	NMOSD serostatus	Free of relapses/relapse reduction (RR)	Follow-up	Disability (EDSS stabilization or improvement)	Safety concerns
Azathioprine (AZA)	Retrospective studies	Off-label (including meta-analysis)	977	AQP4-ab + and -	34–61%	18–47 months	Up to 69% during 5 year	GI, infections, Hepatotoxicity Bone marrow suppression Malignancy
Micophenolate mofetil (MMF)	Retrospective studies	Off-label (including meta-analysis)	799	AQP4-ab + and -	46–73%	20–27 months	Up to 90% during 5 year	GI, infections, Hepatotoxicity Bone marrow suppression Teratogenicity
Rituximab (RTX)	Retrospective studies	Off-label (including meta-analysis)	577	AQP4-ab + and -	62.9 (52–88%)	24–60 months	Up to 97% during 5 year Dif EDSS: -1.16	Mucocutaneous reactions HBV reactivation Severe hypogammaglobulinemia
	Multi-center, randomized, double-blind, placebo-controlled trial (Japan)	Phase 2,3 study UMIN000013453	38 (1:1)	AQP4-ab +	100%	72 weeks	Change in EDSS (− 0.26 vs. − 0.32) NSD	NSD compared with placebo
Tocilizumab (TCZ)	Single-center randomized, open-label, parallel-group study (TANGO; China)	Phase 2,3 study comparing TCZ vs AZA NCT03350633	118 (1:1)	AQP4-ab + and -	TCZ: 92% AZA: 68% RR: NA	48 weeks 48 weeks	OR = 0.34	Pneumonia (3%), herpes zoster virus (2%), deep vein thrombosis (2%), basal ganglia haemorrhage (2%)
	Single-center, open-label trial (China)	Phase 1 and 2 study as monotherapy (completed) NCT03062579	10	AQP4-ab + and -*	NA	1 year	NA	NA
Eculizumab	Multicenter, randomized, placebo-controlled, time-to-event trial (PREVENT trial)	Phase 3 study (add-on therapy) NCT01892345	143 (2:1)	AQP4-ab +	96.1% RR:93.1%	96 weeks	NSD	Increased risk of meningococcal infection. All patients had to receive vaccination 2 weeks prior to first dose
Inebilizumab	Multi-center, randomized, double-blind, placebo-controlled (N-Momentum)	Phase 2/3 study NCT02200770	231 (3:1)	AQP4-ab + and -	87.6% RR: 73% (77.3% in AQP4-ab+)	28 weeks	OR = 0.37	NSD compared to placebo
Satralizumab	Multicenter, randomized, placebo-controlled, time-to-event trial (SakuraSky)	Phase 3 study (add-on therapy) NCT02028884	83 (1:1)	AQP4-ab + and -	AQP4+ 91.5% AQP4-: 56.3% RR:62% (79% in AQP4-ab+)	96 weeks	NA	NSD compared to placebo
	Multicenter, randomized, placebo-controlled, time-to-event trial (SakuraStar)	Phase 3 study (monotherapy) NCT02073279	95 (2:1)	AQP4-ab + and -	AQP4+ 76.5% AQP4-: 63.3% RR:55% (74% in AQP4-ab+)	96 weeks	NA	NSD compared to placebo
Telitacicept	Randomized, placebo-controlled (China)	Phase 3 study (active, recruiting) NCT03330418	118 (estimated enrollment)	AQP4-ab +	NA	Time frame: 144 weeks	NA	NA
Ravulizumab	Multicenter, open-label, external placebo-controlled	Phase 3 study (active, recruiting) NCT04201262	55 (estimated enrollment)	AQP4-ab +*	NA	Time frame: 2 years	NA	NA
Bortezomib	Single-center, Open Label Trial (USA)	Phase 2 add-on study (completed) NCT02893111	5	AQP4-ab+*	NA	Time frame: 1 year	NA	NA
Cetirizine	Single-center, Open Label Trial (USA)	Phase 2 add-on study (completed) NCT02865018	16	AQP4-ab+ (with ON or TM)**	NA	Time frame: 1 year	NA	NA
BAT4406F	Single-center, Open Label Trial (China)	Phase 1 dose-escalation study (not yet recruiting) NCT04146285	48 (estimated enrollment)	AQP4-ab + and -*	NA	NA	NA	NA
SHR1459	Single-center, Open Label Trial (China)	Phase 2 study (not yet recruiting) NCT04670770	10 (estimated enrollment)	AQP4-ab +*	NA	Time frame: 52 weeks	NA	NA
Hematopoietic Stem Cell Transplantation	Single Group Assignment; Open Label study (USA)	Phase 1, 2 study (completed) NCT00787722	13 (12 completed the study)	AQP4-ab+ (n=12) and unknown (n=1)	80%	5 years	at baseline: 4.4 at FU: 3.3	1/13 died due to unrelated complications from SLE Neutropenic fever (5/13), Hypophosphatemia (9/13) Infections (1/13)

*AQP4*-*ab* + positive aquaporin-4 antibodies, *NMOSD* neuromyelitis optica spectrum disorders, *EDSS* Expanded disability Status Scale, *NSD* no statistical differences, *USA* United States of America, *NA* not available, *ON* optic neuritis, *TM* transverse myelitis, *SLE* systemic lupus erythematosus, *GI* gastrointestinal

*Participants must have AQP4-ab + and a diagnosis of NMOSD as defined by the 2015 International Consensus Diagnostic Criteria. ** Diagnosis of NMOSD as defined by the 2006 Revised NMO Diagnostic Criteria

### Long-term relapse prevention treatment: old era

#### Azathioprine and mycophenolate mofetil

For full biologic effects to be observed, azathioprine (AZA) and mycophenolate (MMF) treatment require at least 4–6 months duration. Therefore, oral steroids should also be given, to provide an immunosuppressive bridge from treatment onset [18]. AZA and MMF have demonstrated efficacy in different studies of NMOSD patients, with significant reduction in ARR, and stabilization or improvement of EDSS scores (Table 3) [93–106]. In a prospective RCT comparing AZA to rituximab in NMOSD patients, AZA produced significant decrease in both ARR (54% of patients were relapse-free after 1 year) and in disability, with a drop in EDSS from 2.40 to 1.95 [94]. In a Brazilian study on 150 patients, 69% presented no accumulation of disability after 5-year follow-up [93]. Another multicenter retrospective study with 103 AQP4-ab-positive NMOSD patients showed 89% experienced significant ARR reduction on AZA (1.5 vs. 0.0), and 61% of patients were relapse-free, although no significant reduction in EDSS was reported [95]. Of note, AZA was discontinued in 62% of patients due to side effects ([SEs]; increased liver enzymes and pancytopenia) [95]. Rituximab and MMF were more efficacious than AZA in comparative effectiveness studies [103–105].

One recent study reported 50.7% of patients experienced a relapse on MMF, 59.7% continued on MMF, and 83% showed stabilization or improvement of disability at most recent follow-up [97]. In another study, 9 of 10 patients with ongoing relapse activity on MMF were switched to rituximab, and showed reduction or absence of new relapses [98].

#### Rituximab

In patients treated with rituximab, NMOSD activity has been correlated with B cell levels, but not AQP4-ab levels [106]. Two meta-analyses suggested relevant reduction of ARR with stabilization or improvement in EDSS [100, 102]. Comparative studies have shown rituximab is more effective than AZA and MMF in decreasing ARR and relapse severity as well as preventing new relapses [103–105]. Even when rituximab dosing was not optimal, failure rates were still lower than with AZA or MMF [103]. So far, only one phase 2/3, multi-center, double-blind, RCT (RIN-1) [40] evaluating rituximab in NMOSD patients has been conducted in Japan. Results showed that patients treated with rituximab experienced significantly lower ARR compared to placebo (0% vs. 37%) after 72-week follow-up. No statistical differences were observed in EDSS. However, the small number of patients, varying relapse history, concurrent use of high-dose steroids, and inclusion of AQP4-ab-positive patients only mean results need to be interpreted with caution.

#### Tocilizumab

Two open-label trials with IV tocilizumab in AQP4-ab-positive NMOSD patients, either as concomitant treatment or monotherapy, have demonstrated reduction in ARR [107, 108]. Tocilizumab has also been associated with clinical stabilization in NMOSD patients who failed one or more first-line treatments. Patients who were refractory to rituximab (highly active NMOSD) showed reduction in clinical and radiological activity after treatment with tocilizumab [107]. Of note, tocilizumab improved fatigue and neuropathic pain in a small trial of NMOSD patients, suggesting that the IL-6 pathway may be involved in these mechanisms [108]. Recently, an open-label, multicenter, phase 2 RCT evaluating safety and efficacy of tocilizumab vs. AZA in highly relapsing NMOSD (TANGO) [92] reported a significantly shorter median time to first relapse in the AZA group compared to the tocilizumab group. In addition, 47% of patients treated with AZA and 14% on tocilizumab experienced a relapse at 90 weeks. Moreover, SEs were reported in 83% of patients on AZA and 61% in the tocilizumab group [80]. Additionally, a phase 1/2 single center, open-label trial has been completed to determine if tocilizumab as monotherapy contributes to reduce ARR and improve disability in NMOSD patients who experienced relapses on immunosuppressive therapies (including rituximab). Results are not yet available (Table 3). Subcutaneous tocilizumab treatment has demonstrated similar effectiveness to IV administration [109].

#### Other treatments

Cyclophosphamide, methotrexate, and mitoxantrone, with or without concomitant steroids, were evaluated in uncontrolled retrospective studies [18, 21]. In all but one, disease stabilization was observed. One study reported that cyclophosphamide was ineffective and associated with SE [110].

### Long-term relapse prevention treatment: present era

#### Eculizumab

Efficacy and safety results of IV eculizumab were recently published from a time-to-event RCT (PREVENT trial) [13] conducted in AQP4-ab-positive NMOSD patients, with highly active disease. Relapses were determined by an independent committee and secondary outcomes included ARR, EDSS, and quality of life scales [13, 111]. Patients were randomized 2:1 to receive eculizumab or placebo (plus ongoing immunosuppressive treatment). Nearly 76% of patients were on concomitant immunosuppressive therapy (AZA, MMF, or oral steroids) during the trial [13, 111]. Patients who had received rituximab in the 3 months prior to the study were excluded. Approximately one-quarter confirmed efficacy of eculizumab as monotherapy [13, 111]. NMOSD patients (*n* = 143) receiving eculizumab experienced a relative risk reduction of 94% (hazard ratio [HR]: 0.06; *p* < 0.001) over the 48-week study period, compared to placebo (ARR 3% vs. 46%, respectively) [13]. Although patients on eculizumab had lower adjudicated ARR than placebo (0.02 vs. 0.35; *p* < 0.001), no statistical differences in EDSS or quality of life outcomes were observed between groups. Regarding safety, eculizumab showed a profile similar to that of placebo, even in the open-label extension study recently published [13]. Given the fact that eculizumab increases the risk of infection by encapsulated bacteria, particularly meningococcus, all patients received meningococcal vaccination 2-week prior to the first dose [111]. However, patients on eculizumab remain at risk for meningococcal disease even after receipt of meningococcal vaccines and some health care providers in the USA as well as public health agencies in other countries recommend prophylactic treatment with appropriate antibiotics for the duration of eculizumab treatment [112]. No cases of drug discontinuation or meningococcal infection were reported, although one case of Neisseria gonorrhoeae infection was observed in the open-label study [13].

#### Inebilizumab

Results on efficacy and safety of IV inebilizumab were recently published in a double-blind, RCT phase 2/3 (N-MOmentum trial) [21] in AQP4-ab-positive (*n* = 213) AQP4-ab-negative (*n* = 17), and MOG-ab positive (*n* = 7) NMOSD patients. All patients received concomitant oral steroids to reduce risk of relapse following B cell depletion. Relapses (defined specifically for this study) were confirmed by an adjudication panel (MRI confirmation of relapse was required in some cases). Secondary outcomes included EDSS worsening, change in low-contrast VA score, cumulative MRI lesions, and number of NMOSD-related hospitalizations. Immunosuppressant therapy use was allowed prior to trial with different wash-out periods and other laboratory parameters had to be normalized. Concomitant immunosuppression use was not allowed. Patients were randomized 3:1 to receive inebilizumab or placebo, to mitigate risk in the placebo group [111]. Inclusion criteria were at least 1 relapse within the previous 1 year, or at least 2 relapses within the past 2 years and an EDSS score of 8 or less [21, 111]. Patients who received inebilizumab experienced 73% relative risk reduction (HR: 0.272; *p* < 0.0001) in number of relapses over the 28-week study period compared to placebo (12% vs. 39%, respectively) [21]. This effect was more robust in AQP4-ab-positive patients (11% vs. 42%, respectively). Positive effects on secondary outcomes were also observed, except for low-contrast VA scores [21, 111]. Regarding safety, inebilizumab showed a favorable profile, similar to placebo. No deaths occurred throughout the randomized controlled period, although 2 patients discontinued treatment in the inebilizumab group due to SEs. Two deaths were reported in the open-label extension study, one of them not related to inebilizumab [111].

#### Satralizumab

Results on efficacy and safety of subcutaneous satralizumab were recently published in two, time-to-event RCTs, conducted in AQP4-ab-positive and negative (~ 30% of the study population) NMOSD patients [19, 20]. The first study (Sakura-Sky) [19] was an add-on trial (AZA, MMF, or oral steroids, but not rituximab), and primary outcome was time to first protocol-defined relapse in the double-blind period, as adjudicated by committee blinded to clinical endpoints. Secondary outcomes included change in scale for pain and fatigue (FACIT-F) score [19]. Patients were randomized 1:1 to receive satralizumab or placebo (plus ongoing immunosuppressive treatment). Inclusion criteria required patients to have had at least 2 relapses within the previous 2 years, with one of these occurring within the past year, or at least 1 relapse within the past year. EDSS scores of 6.5 or less were allowed. Seven adolescents were enrolled (4 in the satralizumab and 3 in the placebo group). Patients who received satralizumab experienced a relative risk reduction of 62% in time to first relapse (HR: 0.38; *p* = 0.02; median double-blind treatment duration was 107.4 weeks for satralizumab and 32.5 weeks for placebo) compared to placebo (20% vs. 43%, respectively). AQP4-ab-positive patients showed 79% reduction in relapse risk and AQP4-ab-negative patients 34% [19]. At 96 weeks, 78% of patients receiving satralizumab were relapse-free, compared to 59% receiving placebo. At 48 and 96 weeks, 92% of AQP4-ab-positive patients on satralizumab were relapse-free. No change was observed in pain or fatigue scores from baseline [19, 111].

Satralizumab was also studied as monotherapy in a similar-design phase 3 study (SAkuraStar trial) [20]. Patients were randomized 2:1 to satralizumab or placebo. Unlike the SAkuraSky trial, concomitant immunosuppressant use was not allowed. Inclusion criteria and clinical endpoints were the same as in the SAkuraSky trial. Patients who received satralizumab as monotherapy experienced a relative risk reduction of 55% in time to first relapse (HR: 0.45; *p* = 0.01) compared to the placebo group (30% vs. 50%, respectively) [20]. At 96 weeks, 72% of patients receiving satralizumab were relapse-free, compared to 51% of patients on placebo. The response was more robust in the AQP4-ab-positive patients (77% vs. 41%) [20, 111].

Satralizumab showed a favorable safety profile in both studies. No death or anaphylactic reactions were observed. Only one patient in the SAkuraStar trial discontinued treatment due to pneumonia [19, 20, 111].

### Long-term relapse prevention treatment: future era

New drugs including BAT4406F, SHR1459, ravulizumab, bortezomib, cetirizine, telitacicept, and autologous hematopoietic stem cell transplantation (HSCT) are currently under investigation in ongoing RCTs (Tables 1 and 3).

A phase I RCT on safety, tolerability, and pharmacokinetics of BAT4406F (a fully humanized anti-CD20 monoclonal antibody) through intravenous infusion will be starting soon in NMOSD patients (NCT04146285), as will an open-label phase 2 trial evaluating efficacy and safety of SHR1459 (Bruton’s Tyrosine Kinase Inhibitor) in (NCT04670770).

Ravulizumab, a molecule derived from eculizumab, is a second generation anti-C5 complement protein, with an extended serum half-life (three- to four-fold) [113]. A phase 3, external placebo-controlled, open-label, multicenter study evaluating efficacy and safety of ravulizumab in NMOSD patients is currently underway (NCT04201262).

Bortezomib, a proteasome inhibitor used in the treatment of multiple myeloma, has been studied in a cohort of Chinese patients [114]. Four out of 5 patients receiving bortezomib as rescue therapy were relapse-free at 1 year. Stabilization was associated with decrease in serum AQP4-ab titers, as well as in peripheral plasma cell and precursor B cell counts [114]. Phase 2 has been completed, but results are not yet available.

A small pilot study on cetirizine (a second-generation antihistamine) as add-on therapy reduced ARR at 1-year of follow-up, although no significant difference in EDSS scores were observed [115]. Further research will be needed to confirm these results.

An ongoing phase 3 randomized, placebo-controlled study is evaluating telitacicept, an inhibitor of B lymphocyte stimulator (BlyS) and of APRIL [116], in AQP4-ab-positive NMOSD patients without recent immunosuppressive treatment (NCT03330418). This drug was recently approved for treatment of lupus, after showing efficacy and safety in a pivotal phase 2b trial (NCT02885610).

Another study showed prolonged drug-free remission in 11 NMOSD patients with seroconversion of positive AQP4-ab status to negative, following non-myeloablative autologous hematopoietic stem cell transplant HSCT [117]. Most recently, a meta-analysis evaluating autologous HSCT in 31 NMOSD patients showed 76% progression-free survival and 0% transplant-related mortality in treated patients [118].

Finally, aquaporumab a targeted non-immunosuppressive therapy has shown effects in AQP4-ab-positive NMOSD cell cultures [119].

### Therapeutic considerations in pregnancy and pediatric NMOSD patients

Trophoblasts in the placenta express AQP4 and are exposed to maternal blood containing AQP4-ab [120]. Several studies have shown women with NMOSD are at increased risk of relapse, particularly postpartum [121, 122]. However, available data is insufficient to precisely define risk of relapse during pregnancy [121–125]. MMF, methotrexate, or mitoxantrone are contraindicated in pregnant women. AZA, rituximab, eculizumab, and steroids appear to be relatively safe and may be continued, depending on disease severity [123–125]. Additionally, tocilizumab may be also be an option in pregnant women with severe NMOSD [125].

Previous studies have identified relapsing NMOSD in pediatric patients [126, 127]. Although the list of new treatments for adults with NMOSD is increasing, only one trial (Sakura-Sky) [19] has included pediatric patients (over the age of 12). Pediatric NMOSD patients should be prescribed immunosuppressive treatment. Until more experience is gained with newer agents, therapeutic options available for this age group include rituximab, AZA, or MMF [127].

## Conclusions and future perspectives

During the last two decades, knowledge on the pathophysiological mechanisms involved in NMOSD has advanced significantly. In addition, new diagnostic features have been described, opening the door to new therapeutic targets. A clear demonstration of this, are the 3 new monoclonal antibodies, targeting 3 different disease pathways, showing efficacy in recent phase III-controlled trials [13, 19–21]. Although this has shifted broad immunosuppression to more narrow treatment targets, unmet needs persist in NMOSD patients. Therapies improving regeneration and restoring functionality are missing, and AQP4-ab-negative patients are underrepresented or absent from most clinical trials, so that confirmation of the underlying disease mechanism has not been possible in this particular patient group [19, 20]. Thus, the absence of observed efficacy in AQP4-ab-negative NMOSD patients may be attributable to the greater degree of disease heterogeneity within the general AQP4-ab-negative subpopulation [18–20]. These findings could be also interpreted as that some AQP4-ab-negative patients with clinical and neuroradiological features of NMOSD have a different underlying antibody target [10]. This could be partially explained by the presence of MOG-ab in a subgroup of the AQP4-ab-negative patients [15, 21]. Although AQP4-ab-negative patients are considered in the 2015 NMOSD diagnostic criteria [10], a large diagnostic disagreement was reported in this subgroup of patients even among experts in this field [128], since the criteria were not consistently used.

Long-term impact of recently developed drugs remains to be established. Which is the best drug to initiate treatment? Does aggressiveness of disease condition drug selection? Is there really one compound that is more effective than another? How do we evaluate suboptimal response to treatment? These are all unanswered questions. In MS, some clarity on these issues has been achieved. Although desirable, head-to-head superiority studies involving different drugs are currently underway; they are difficult to complete given the low prevalence of disease, paucity of relapses in treated patients, and the heterogeneity of the study populations. Hopefully, prospective multi-center, real-life studies will provide high-level evidence-based information, on the best treatment regimens, as well as their long-term effects. Finally, can long-term treatment be discontinued in the absence of disease activity? This is a critical problem for patients who have to weigh life-long immunosuppressive treatment, against risk of relapse and the burden of disability. Currently, there is no consensus regarding the optimum duration of long-term preventive treatment, and therefore a frequent clinical dilemma is the feasibility of treatment withdrawal in patients who have achieved a sustained period of clinical stability. However, immunosuppressant therapies discontinuation may increase the risk of relapse in AQP4-ab-positive NMOSD patients even after 5 years of remission [129]. For this reason, induction of immune tolerance [130], although a very recent concept, is a fascinating new alternative well worth exploring to avoid the need for long-term drug administration in NMOSD patients.

## Data Availability

The datasets generated and/or analyzed during the current study are not publicly available but are available from the corresponding author upon reasonable request.

## Abbreviations

AQP4: Aquaporin-4
CNS: Central nervous system
ARR: Annualized relapse rates
RCTs: Randomized clinical trials
OAPs: Orthogonal arrays of particles
AZA: Azathioprine
MMF: Mycophenolate
ADEM: Acute disseminated encephalomyelitis
GFAP: Glial fibrillary acidic protein
MAC: Membrane attack complex
CSF: Cerebrospinal fluid
Gd: Gadolinium
BBB: Blood-brain barrier
BAFF: B cell activating factor
APRIL: Proliferation-inducing ligand
CDC: Complement-dependent cytotoxicity
ADCC: Antibody-dependent cellular cytotoxicity
EAAT2: Excitatory amino acid transporter 2
PNMs: Polymorphonuclear leucocytes
ECP: Eosinophil cationic protein
EDN: Eosinophil-derived neurotoxin
EPX: Eosinophil peroxidase
MBP: Major basic protein
IgG: Immunoglobulin G
IgM: Immunoglobulin M
IVMP: Intravenous methylprednisolone
LETM: Longitudinally extensive transverse myelitis
MOG: Myelin oligodendrocyte glycoprotein
MRI: Magnetic resonance imaging
MS: Multiple sclerosis
NMO: Neuromyelitis optica
NMOSD: NMO spectrum disorder
OCB: Oligoclonal IgG bands
ON: Optic neuritis
TM: transverse myelitis
APS: Area postrema syndrome
PEX: Plasma exchange
IA: Immunoadsorption
SEs: Side effects
VA: Visual acuity
HSCT: Autologous hematopoietic stem cell transplantation

## References

[1] Kawachi I, Lassmann H (2017). Neurodegeneration in multiple sclerosis and neuromyelitis optica. J Neurol Neurosurg Psychiatry..

[2] Miyazawa I, Fujihara K, Itoyama Y (2002). Eugène Devic (1858-1930). J Neurol..

[3] Wingerchuk DM, Hogancamp WF, O'Brien PC, Weinshenker BG (1999). The clinical course of neuromyelitis optica (Devic's syndrome). Neurology..

[4] Lennon VA, Wingerchuk DM, Kryzer TJ, et al. A serum autoantibody marker of neuromyelitis optica: distinction from multiple sclerosis. Lancet. 2004;364(9451):2106-12. 10.1016/S0140-6736(04)17551-X 15589308

[5] Lennon VA, Kryzer TJ, Pittock SJ (2005). IgG marker of optic-spinal multiple sclerosis binds to the aquaporin-4 water channel. J Exp Med..

[6] Wingerchuk DM, Lennon VA, Pittock SJ, Lucchinetti CF, Weinshenker BG (2006). Revised diagnostic criteria for neuromyelitis optica. Neurology..

[7] Wingerchuk DM, Lennon VA, Lucchinetti CF, Pittock SJ, Weinshenker BG (2007). The spectrum of neuromyelitis optica. Lancet Neurol..

[8] Miller DH, Weinshenker BG, Filippi M (2008). Differential diagnosis of suspected multiple sclerosis: a consensus approach. Mult Scler..

[9] Mader S, Gredler V, Schanda K (2011). Complement activating antibodies to myelin oligodendrocyte glycoprotein in neuromyelitis optica and related disorders. J Neuroinflammation..

[10] Wingerchuk DM, Banwell B, Bennett JL (2015). International Panel for NMO Diagnosis. International consensus diagnostic criteria for neuromyelitis optica spectrum disorders. Neurology..

[11] Thompson AJ, Banwell BL, Barkhof F, Carroll WM, Coetzee T, Comi G, Correale J, Fazekas F, Filippi M, Freedman MS, Fujihara K, Galetta SL, Hartung HP, Kappos L, Lublin FD, Marrie RA, Miller AE, Miller DH, Montalban X, Mowry EM, Sorensen PS, Tintoré M, Traboulsee AL, Trojano M, Uitdehaag BMJ, Vukusic S, Waubant E, Weinshenker BG, Reingold SC, Cohen JA (2018). Diagnosis of multiple sclerosis: 2017 revisions of the McDonald criteria. Lancet Neurol..

[12] Jarius S, Paul F, Aktas O, Asgari N, Dale RC, de Seze J, Franciotta D, Fujihara K, Jacob A, Kim HJ, Kleiter I, Kümpfel T, Levy M, Palace J, Ruprecht K, Saiz A, Trebst C, Weinshenker BG, Wildemann B (2018). MOG encephalomyelitis: international recommendations on diagnosis and antibody testing. J Neuroinflammation..

[13] Pittock SJ, Berthele A, Fujihara K (2019). Eculizumab in Aquaporin-4-positive neuromyelitis optica spectrum disorder. N Engl J Med..

[14] Höftberger R, Guo Y, Flanagan EP, Lopez-Chiriboga AS, Endmayr V, Hochmeister S, Joldic D, Pittock SJ, Tillema JM, Gorman M, Lassmann H, Lucchinetti CF (2020). The pathology of central nervous system inflammatory demyelinating disease accompanying myelin oligodendrocyte glycoprotein autoantibody. Acta Neuropathol..

[15] Sato DK, Callegaro D, Lana-Peixoto MA (2014). Distinction between MOG antibody-positive and AQP4 antibody-positive NMO spectrum disorders. Neurology..

[16] Reindl M, Waters P (2019). Myelin oligodendrocyte glycoprotein antibodies in neurological disease. Nat Rev Neurol..

[17] Palace J, Lin DY, Zeng D (2019). Outcome prediction models in AQP4-IgG positive neuromyelitis optica spectrum disorders. Brain..

[18] Carnero Contentti E, Rojas JI, Cristiano E (2020). Latin American consensus recommendations for management and treatment of neuromyelitis optica spectrum disorders in clinical practice. Mult Scler Relat Disord..

[19] Yamamura T, Kleiter I, Fujihara K, Palace J, Greenberg B, Zakrzewska-Pniewska B, Patti F, Tsai CP, Saiz A, Yamazaki H, Kawata Y, Wright P, de Seze J (2019). Trial of Satralizumab in neuromyelitis optica spectrum disorder. N Engl J Med..

[20] Traboulsee A, Greenberg BM, Bennett JL (2020). Safety and efficacy of satralizumab monotherapy in neuromyelitis optica spectrum disorder: a randomised, double-blind, multicentre, placebo-controlled phase 3 trial. Lancet Neurol..

[21] Cree BAC, Bennett JL, Kim HJ (2019). N-MOmentum study investigators. Inebilizumab for the treatment of neuromyelitis optica spectrum disorder (N-MOmentum): a double-blind, randomised placebo-controlled phase 2/3 trial. Lancet..

[22] Jung JS, Bhat RV, Preston GM, Guggino WB, Baraban JM, Agre P (1994). Molecular characterization of an aquaporin cDNA from brain: candidate osmoreceptor and regulator of water balance. Proc Natl Acad Sci U S A..

[23] Hinson SR, Romero MF, Popescu BF (2012). Molecular outcomes of neuromyelitis optica (NMO)-IgG binding to aquaporin-4 in astrocytes. Proc Natl Acad Sci U S A..

[24] Crane JM, Lam C, Rossi A (2011). Binding affinity and specificity of neuromyelitis optica autoantibodies to aquaporin-4 M1/M23 isoforms and orthogonal arrays. J Biol Chem..

[25] Papadopoulos MC, Verkman AS (2012). Aquaporin 4 and neuromyelitis optica. Lancet Neurol..

[26] Phuan PW, Ratelade J, Rossi A, Tradtrantip L, Verkman AS (2012). Complement-dependent cytotoxicity in neuromyelitis optica requires aquaporin-4 protein assembly in orthogonal arrays. J Biol Chem..

[27] Popescu BF, Lucchinetti CF (2012). Pathology of demyelinating diseases. Annu Rev Pathol..

[28] Hinson SR, Pittock SJ, Lucchinetti CF (2007). Pathogenic potential of IgG binding to water channel extracellular domain in neuromyelitis optica. Neurology..

[29] Lucchinetti CF, Mandler RN, McGavern D, Bruck W, Gleich G, Ransohoff RM, Trebst C, Weinshenker B, Wingerchuk D, Parisi JE, Lassmann H (2002). A role for humoral mechanisms in the pathogenesis of Devic's neuromyelitis optica. Brain..

[30] Jarius S, Aboul-Enein F, Waters P (2008). Antibody to aquaporin-4 in the long-term course of neuromyelitis optica. Brain..

[31] Jarius S, Franciotta D, Paul F, Bergamaschi R, Rommer PS, Ruprecht K, Ringelstein M, Aktas O, Kristoferitsch W, Wildemann B (2012). Testing for antibodies to human aquaporin-4 by ELISA: sensitivity, specificity, and direct comparison with immunohistochemistry. J Neurol Sci..

[32] Kim W, Lee JE, Li XF, Kim SH, Han BG, Lee BI, Kim JK, Choi K, Kim HJ (2012). Quantitative measurement of anti-aquaporin-4 antibodies by enzyme-linked immunosorbent assay using purified recombinant human aquaporin-4. Mult Scler..

[33] Jarius S, Ruprecht K, Wildemann B, Kuempfel T, Ringelstein M, Geis C, Kleiter I, Kleinschnitz C, Berthele A, Brettschneider J, Hellwig K, Hemmer B, Linker RA, Lauda F, Mayer CA, Tumani H, Melms A, Trebst C, Stangel M, Marziniak M, Hoffmann F, Schippling S, Faiss JH, Neuhaus O, Ettrich B, Zentner C, Guthke K, Hofstadt-van Oy U, Reuss R, Pellkofer H, Ziemann U, Kern P, Wandinger KP, Then Bergh F, Boettcher T, Langel S, Liebetrau M, Rommer PS, Niehaus S, Münch C, Winkelmann A, Zettl U UK, Metz I, Veauthier C, Sieb JP, Wilke C, Hartung HP, Aktas O, Paul F (2012). Contrasting disease patterns in seropositive and seronegative neuromyelitis optica: A multicentre study of 175 patients. J Neuroinflammation..

[34] Weinshenker BG, Wingerchuk DM, Vukusic S (2006). Neuromyelitis optica IgG predicts relapse after longitudinally extensive transverse myelitis. Ann Neurol..

[35] Pittock SJ, Lennon VA, Krecke K (2006). Brain abnormalities in neuromyelitis optica. Arch Neurol..

[36] Matiello M, Schaefer-Klein J, Sun D, Weinshenker BG (2013). Aquaporin 4 expression and tissue susceptibility to neuromyelitis optica. JAMA Neurol..

[37] Kim SH, Kim W, Huh SY (2013). Clinical efficacy of plasmapheresis in patients with neuromyelitis optica spectrum disorder and effects on circulating anti-aquaporin-4 antibody levels. J Clin Neurol..

[38] Bonnan M, Valentino R, Olindo S, et al. Plasma exchange in severe spinal attacks associated with neuromyelitis optica spectrum disorder. Mult Scler. 2009;(4):487–92. 10.1177/1352458508100837 19324982

[39] Kim SH, Kim W, Li XF (2011). Repeated treatment with rituximab based on the assessment of peripheral circulating memory B cells in patients with relapsing neuromyelitis optica over 2 years. Arch Neurol..

[40] Tahara M, Oeda T, Okada K (2020). Safety and efficacy of rituximab in neuromyelitis optica spectrum disorders (RIN-1 study): a multicentre, randomised, double-blind, placebo-controlled trial. Lancet Neurol..

[41] Ratelade J, Bennett JL, Verkman AS (2011). Intravenous neuromyelitis optica autoantibody in mice targets aquaporin-4 in peripheral organs and area postrema. PLoS One..

[42] Asavapanumas N, Ratelade J, Verkman AS. Unique neuromyelitis optica pathology produced in naïve rats by intracerebral administration of NMO-IgG. Acta Neuropathol Comm .2014; 2:48 10.1007/s00401-013-1204-8 PMC3954950 24190619

[43] Klawiter EC, Alvarez E, Xu J (2009). NMO-IgG detected in CSF in seronegative neuromyelitis optica. Neurology..

[44] Chihara N, Aranami T, Sato W, Miyazaki Y, Miyake S, Okamoto T, Ogawa M, Toda T, Yamamura T (2011). Interleukin 6 signaling promotes anti-aquaporin 4 autoantibody production from plasmablasts in neuromyelitis optica. Proc Natl Acad Sci U S A..

[45] Bennett JL, Lam C, Kalluri SR (2009). Intrathecal pathogenic anti-aquaporin-4 antibodies in early neuromyelitis optica. Ann Neurol..

[46] Lindner M, Klotz L, Wiendl H (2018). Mechanisms underlying lesion development and lesion distribution in CNS autoimmunity. J Neurochem..

[47] Broadwell RD, Sofroniew MV (1993). Serum proteins bypass the blood-brain fluid barriers for extracellular entry to the central nervous system. Exp Neurol..

[48] Bennett JL, O'Connor KC, Bar-Or A, Zamvil SS, Hemmer B, Tedder TF, von Büdingen HC, Stuve O, Yeaman MR, Smith TJ, Stadelmann C (2015). B lymphocytes in neuromyelitis optica. Neurol Neuroimmunol Neuroinflamm..

[49] Graf J, Mares J, Barnett M, Aktas O, Albrecht P, Zamvil SS, Hartung HP (2020). Targeting B cells to modify MS, NMOSD, and MOGAD: Part 2. Neurol Neuroimmunol Neuroinflamm..

[50] Chihara N, Aranami T, Oki S, Matsuoka T, Nakamura M, Kishida H, Yokoyama K, Kuroiwa Y, Hattori N, Okamoto T, Murata M, Toda T, Miyake S, Yamamura T (2013). Plasmablasts as migratory IgG-producing cells in the pathogenesis of neuromyelitis optica. PLoS One..

[51] Vaknin-Dembinsky A, Brill L, Orpaz N (2010). Preferential increase of B-cell activating factor in the cerebrospinal fluid of neuromyelitis optica in a white population. Mult Scler..

[52] Kaneko K, Sato DK, Nakashima I, Ogawa R, Akaishi T, Takai Y, Nishiyama S, Takahashi T, Misu T, Kuroda H, Tanaka S, Nomura K, Hashimoto Y, Callegaro D, Steinman L, Fujihara K, Aoki M (2018). CSF cytokine profile in MOG-IgG+ neurological disease is similar to AQP4-IgG+ NMOSD but distinct from MS: a cross-sectional study and potential therapeutic implications. J Neurol Neurosurg Psychiatry..

[53] Molnarfi N, Schulze-Topphoff U, Weber MS (2013). MHC class II-dependent B cell APC function is required for induction of CNS autoimmunity independent of myelin-specific antibodies. J Exp Med..

[54] Deenick EK, Chan A, Ma CS, Gatto D, Schwartzberg PL, Brink R, Tangye SG (2010). Follicular helper T cell differentiation requires continuous antigen presentation that is independent of unique B cell signaling. Immunity..

[55] Fujihara K, Bennett JL, de Seze J (2020). Interleukin-6 in neuromyelitis optica spectrum disorder pathophysiology. Neurol Neuroimmunol Neuroinflamm..

[56] Quan C, Yu H, Qiao J (2013). Impaired regulatory function and enhanced intrathecal activation of B cells in neuromyelitis optica: distinct from multiple sclerosis. Mult Scler..

[57] Varrin-Doyer M, Spencer CM, Schulze-Topphoff U (2012). Aquaporin 4-specific T cells in neuromyelitis optica exhibit a Th17 bias and recognize Clostridium ABC transporter. Ann Neurol..

[58] Bradl M, Misu T, Takahashi T, Watanabe M, Mader S, Reindl M, Adzemovic M, Bauer J, Berger T, Fujihara K, Itoyama Y, Lassmann H (2009). Neuromyelitis optica: pathogenicity of patient immunoglobulin in vivo. Ann Neurol..

[59] Brum DG, Barreira AA, dos Santos AC (2010). HLA-DRB association in neuromyelitis optica is different from that observed in multiple sclerosis. Mult Scler..

[60] Deschamps R, Paturel L, Jeannin S (2011). Different HLA class II (DRB1 and DQB1) alleles determine either susceptibility or resistance to NMO and multiple sclerosis among the French Afro-Caribbean population. Mult Scler..

[61] Linhares UC, Schiavoni PB, Barros PO (2013). The ex vivo production of IL-6 and IL-21 by CD4+ T cells is directly associated with neurological disability in neuromyelitis optica patients. J Clin Immunol..

[62] Kebir H, Kreymborg K, Ifergan I (2007). Human TH17 lymphocytes promote blood-brain barrier disruption and central nervous system inflammation. Nat Med..

[63] Korn T, Mitsdoerffer M, Croxford AL (2008). IL-6 controls Th17 immunity in vivo by inhibiting the conversion of conventional T cells into Foxp3+ regulatory T cells. Proc Natl Acad Sci U S A..

[64] Zipfel PF, Skerka C (2009). Complement regulators and inhibitory proteins. Nat Rev Immunol..

[65] Vincent T, Saikali P, Cayrol R (2008). Functional consequences of neuromyelitis optica-IgG astrocyte interactions on blood-brain barrier permeability and granulocyte recruitment. J Immunol..

[66] Jarius S, Paul F, Franciotta D (2011). Cerebrospinal fluid findings in aquaporin-4 antibody positive neuromyelitis optica: results from 211 lumbar punctures. J Neurol Sci..

[67] Ratelade J, Zhang H, Saadoun S, Bennett JL, Papadopoulos MC, Verkman AS (2012). Neuromyelitis optica IgG and natural killer cells produce NMO lesions in mice without myelin loss. Acta Neuropathol..

[68] Tradtrantip L, Yao X, Su T (2017). Bystander mechanism for complement-initiated early oligodendrocyte injury in neuromyelitis optica. Acta Neuropathol..

[69] Duan T, Smith AJ, Verkman AS (2018). Complement-dependent bystander injury to neurons in AQP4-IgG seropositive neuromyelitis optica. J Neuroinflammation..

[70] Hinson SR, Roemer SF, Lucchinetti CF (2008). Aquaporin-4-binding autoantibodies in patients with neuromyelitis optica impair glutamate transport by down-regulating EAAT2. J Exp Med..

[71] Lucchinetti CF, Guo Y, Popescu BF, Fujihara K, Itoyama Y, Misu T (2014). The pathology of an autoimmune astrocytopathy: lessons learned from neuromyelitis optica. Brain Pathol..

[72] Howe CL, Kaptzan T, Magaña SM (2014). Neuromyelitis optica IgG stimulates an immunological response in rat astrocyte cultures. Glia..

[73] Chen T, Lennon VA, Liu YU (2020). Astrocyte-microglia interaction drives evolving neuromyelitis optica lesion. J Clin Invest..

[74] Dejanovic B, Huntley MA, De Mazière A, et al. Changes in the synaptic proteome in tauopathy and rescue of tau-induced synapse loss by C1q antibodies. Neuron. 2018;100(6):1322-1336.e7. 10.1016/j.neuron.2018.10.014 30392797

[75] Ten VS, Yao J, Ratner V (2010). Complement component c1q mediates mitochondria-driven oxidative stress in neonatal hypoxic-ischemic brain injury. J Neurosci..

[76] Winkler A, Wrzos C, Haberl M, Weil MT, Gao M, Möbius W, Odoardi F, Thal DR, Chang M, Opdenakker G, Bennett JL, Nessler S, Stadelmann C (2021). Blood-brain barrier resealing in neuromyelitis optica occurs independently of astrocyte regeneration. J Clin Invest..

[77] Herges K, de Jong BA, Kolkowitz I, Dunn C, Mandelbaum G, Ko RM, Maini A, Han MH, Killestein J, Polman C, Goodyear AL, Dunn J, Steinman L, Axtell RC (2012). Protective effect of an elastase inhibitor in a neuromyelitis optica-like disease driven by a peptide of myelin oligodendroglial glycoprotein. Mult Scler..

[78] Saadoun S, Waters P, MacDonald C, Bell BA, Vincent A, Verkman AS, Papadopoulos MC (2012). Neutrophil protease inhibition reduces neuromyelitis optica-immunoglobulin G-induced damage in mouse brain. Ann Neurol..

[79] Acharya KR, Ackerman SJ (2014). Eosinophil granule proteins: form and function. J Biol Chem..

[80] Zhang C, Zhang M, Qiu W (2020). Safety and efficacy of tocilizumab versus azathioprine in highly relapsing neuromyelitis optica spectrum disorder (TANGO): an open-label, multicentre, randomised, phase 2 trial. Lancet Neurol..

[81] Songthammawat T, Srisupa-Olan T, Siritho S (2019). A pilot study comparing treatments for severe attacks of neuromyelitis optica spectrum disorders: Intravenous methylprednisolone (IVMP) with add-on plasma exchange (PLEX) versus simultaneous ivmp and PLEX. Mult Scler Relat Disord..

[82] Abboud H, Petrak A, Mealy M (2016). Treatment of acute relapses in neuromyelitis optica: Steroids alone versus steroids plus plasma exchange. Mult Scler..

[83] Kleiter I, Gahlen A, Borisow N, Fischer K, Wernecke KD, Wegner B, Hellwig K, Pache F, Ruprecht K, Havla J, Krumbholz M, Kümpfel T, Aktas O, Hartung HP, Ringelstein M, Geis C, Kleinschnitz C, Berthele A, Hemmer B, Angstwurm K, Stellmann JP, Schuster S, Stangel M, Lauda F, Tumani H, Mayer C, Zeltner L, Ziemann U, Linker R, Schwab M, Marziniak M, Then Bergh F, Hofstadt-van Oy U, Neuhaus O, Winkelmann A, Marouf W, Faiss J, Wildemann B, Paul F, Jarius S, Trebst C, on behalf of the Neuromyelitis Optica Study Group (2016). Neuromyelitis optica: evaluation of 871 attacks and 1,153 treatment courses. Ann Neurol..

[84] Stiebel-Kalish H, Hellmann MA (2019). Does time equal vision in the acute treatment of a cohort of AQP4 and MOG optic neuritis?. Neurol Neuroimmunol Neuroinflamm..

[85] Kleiter I, Gahlen A, Borisow N, et al; NEMOS (Neuromyelitis Optica Study Group). Apheresis therapies for NMOSD attacks: a retrospective study of 207 therapeutic interventions. Neurol Neuroimmunol Neuroinflamm. 2018;5(6):e504. 10.1212/NXI.0000000000000504 PMC6192689 30345331

[86] Bonnan M, Valentino R, Debeugny S, Merle H, Fergé JL, Mehdaoui H, Cabre P (2018). Short delay to initiate plasma exchange is the strongest predictor of outcome in severe attacks of NMO spectrum disorders. J Neurol Neurosurg Psychiatry..

[87] Elsone L, Panicker J, Mutch K, Boggild M, Appleton R, Jacob A (2014). Role of intravenous immunoglobulin in the treatment of acute relapses of neuromyelitis optica: experience in 10 patients. Mult Scler..

[88] Absoud M, Brex P, Ciccarelli O, Diribe O, Giovannoni G, Hellier J, Howe R, Holland R, Kelly J, McCrone P, Murphy C, Palace J, Pickles A, Pike M, Robertson N, Jacob A, Lim M (2017). A multicentre randomiSed controlled TRial of IntraVEnous immunoglobulin compared with standard therapy for the treatment of transverse myelitis in adults and children (STRIVE). Health Technol Assess..

[89] Li X, Tian DC, Fan M, Xiu Y, Wang X, Li T, Jia D, Xu W, Song T, Shi FD, Zhang X (2020). Intravenous immunoglobulin for acute attacks in neuromyelitis optica spectrum disorders (NMOSD). Mult Scler Relat Disord..

[90] Mealy MA, Shin K, John G, Levy M (2015). Bevacizumab is safe in acute relapses of neuromyelitis optica. Clin Exp Neuroimmunol..

[91] Fox E, Lovett-Racke AE, Gormley M (2021). A phase 2 multicenter study of ublituximab, a novel glycoengineered anti-CD20 monoclonal antibody, in patients with relapsing forms of multiple sclerosis. Mult Scler..

[92] Mealy MA, Levy M (2019). A pilot safety study of ublituximab, a monoclonal antibody against cd20, in acute relapses of neuromyelitis optica spectrum disorder. Medicine.

[93] Bichuetti DB, Perin MMM, Souza NA, Oliveira EML (2019). Treating neuromyelitis optica with azathioprine: 20-year clinical practice. Mult Scler..

[94] Nikoo Z, Badihian S, Shaygannejad V, Asgari N, Ashtari F (2017). Comparison of the efficacy of azathioprine and rituximab in neuromyelitis optica spectrum disorder: a randomized clinical trial. J Neurol..

[95] Elsone L, Kitley J, Luppe S (2014). Long-term efficacy, tolerability and retention rate of azathioprine in 103 aquaporin-4 antibody-positive neuromyelitis optica spectrum disorder patients: a multicentre retrospective observational study from the UK. Mult Scler..

[96] Espiritu AI, Pasco PMD (2019). Efficacy and tolerability of azathioprine for neuromyelitis optica spectrum disorder: a systematic review and meta-analysis. Mult Scler Relat Disord..

[97] Montcuquet A, Collongues N, Papeix C, Zephir H, Audoin B, Laplaud D, Bourre B, Brochet B, Camdessanche JP, Labauge P, Moreau T, Brassat D, Stankoff B, de Seze J, Vukusic S, Marignier R, on behalf of NOMADMUS study group and the Observatoire Français de la Sclérose en Plaques (OFSEP) (2017). Effectiveness of mycophenolate mofetil as first-line therapy in AQP4-IgG, MOG-IgG, and seronegative neuromyelitis optica spectrum disorders. Mult Scler..

[98] Huh SY, Kim SH, Hyun JW (2014). Mycophenolate mofetil in the treatment of neuromyelitis optica spectrum disorder. JAMA Neurol..

[99] Songwisit S, Kosiyakul P, Jitprapaikulsan J, Prayoonwiwat N, Ungprasert P, Siritho S (2020). Efficacy and safety of mycophenolate mofetil therapy in neuromyelitis optica spectrum disorders: a systematic review and meta-analysis. Sci Rep..

[100] Gao F, Chai B, Gu C, Wu R, Dong T, Yao Y, Zhang Y (2019). Effectiveness of rituximab in neuromyelitis optica: a meta-analysis. BMC Neurol..

[101] Ciron J, Audoin B, Bourre B, et al; NOMADMUS group, under the aegis of OFSEP, SFSEP. Recommendations for the use of Rituximab in neuromyelitis optica spectrum disorders. Rev Neurol (Paris). 2018;174(4):255-264. 10.1016/j.neurol.2017.11.005 29606320

[102] Damato V, Evoli A, Iorio R (2016). Efficacy and safety of Rituximab therapy in neuromyelitis optica spectrum disorders: a systematic review and meta-analysis. JAMA Neurol..

[103] Mealy MA, Wingerchuk DM, Palace J (2014). Comparison of relapse and treatment failure rates among patients with neuromyelitis optica: Multicenter study of treatment efficacy. JAMA Neurol.

[104] Jeong IH, Park B, Kim SH, Hyun JW, Joo J, Kim HJ (2016). Comparative analysis of treatment outcomes in patients with neuromyelitis optica spectrum disorder using multifaceted endpoints. Mult Scler..

[105] Stellmann JP, Krumbholz M, Friede T, Gahlen A, Borisow N, Fischer K (2017). Immunotherapies in neuromyelitis optica spectrum disorder: efficacy and predictors of response. J Neurol Neurosurg Psychiatry.

[106] Pellkofer HL, Krumbholz M, Berthele A, Hemmer B, Gerdes LA, Havla J, Bittner R, Canis M, Meinl E, Hohlfeld R, Kuempfel T (2011). Long-term follow-up of patients with neuromyelitis optica after repeated therapy with rituximab. Neurology..

[107] Ringelstein M, Ayzenberg I, Harmel J, Lauenstein AS, Lensch E, Stögbauer F, Hellwig K, Ellrichmann G, Stettner M, Chan A, Hartung HP, Kieseier B, Gold R, Aktas O, Kleiter I (2015). Long-term therapy with interleukin 6 receptor blockade in highly active neuromyelitis optica spectrum disorder. JAMA Neurol..

[108] Araki M, Matsuoka T, Miyamoto K, Kusunoki S, Okamoto T, Murata M, Miyake S, Aranami T, Yamamura T (2014). Efficacy of the anti-IL-6 receptor antibody tocilizumab in neuromyelitis optica: a pilot study. Neurology..

[109] Lotan I, Charlson RW, Ryerson LZ (2019). Effectiveness of subcutaneous tocilizumab in neuromyelitis optica spectrum disorders. Mult Scler Relat Disord..

[110] Bichuetti DB, Oliveira EM, Boulos Fde C, Gabbai AA (2012). Lack of response to pulse cyclophosphamide in neuromyelitis optica: evaluation of 7 patients. Arch Neurol..

[111] Levy M, Fujihara K, Palace J (2021). New therapies for neuromyelitis optica spectrum disorder. Lancet Neurol..

[112] McNamara LA, Topaz N, Wang X, Hariri S, Fox L, MacNeil JR (2017). High Risk for Invasive Meningococcal Disease Among Patients Receiving Eculizumab (Soliris) Despite Receipt of Meningococcal Vaccine. MMWR Morb Mortal Wkly Rep..

[113] Peffault de Latour R, Brodsky RA, Ortiz S, Risitano AM (2020). Pharmacokinetic and pharmacodynamic effects of ravulizumab and eculizumab on complement component 5 in adults with paroxysmal nocturnal haemoglobinuria: results of two phase 3 randomised, multicentre studies. Br J Haematol..

[114] Zhang C, Tian DC, Yang CS (2017). Safety and efficacy of Bortezomib in patients with highly relapsing neuromyelitis optica spectrum disorder. JAMA Neurol..

[115] Katz Sand I, Fabian MT, Telford R, Kraus TA, Chehade M, Masilamani M, Moran T, Farrell C, Ebel S, Cook LJ, Rose J, Lublin FD (2018). Open-label, add-on trial of cetirizine for neuromyelitis optica. Neurol Neuroimmunol Neuroinflamm..

[116] Liossis SN, Staveri C. What's new in the treatment of systemic lupus erythematosus. Front Med (Lausanne). 2021;8:655100. 10.3389/fmed.2021.655100 PMC7973110 33748165

[117] Burt RK, Balabanov R, Han X, Burns C, Gastala J, Jovanovic B, Helenowski I, Jitprapaikulsan J, Fryer JP, Pittock SJ (2019). Autologous nonmyeloablative hematopoietic stem cell transplantation for neuromyelitis optica. Neurology..

[118] Zhang P, Liu B (2020). Effect of autologous hematopoietic stem cell transplantation on multiple sclerosis and neuromyelitis optica spectrum disorder: a PRISMA-compliant meta-analysis. Bone Marrow Transplant..

[119] Duan T, Tradtrantip L, Phuan PW (2020). Affinity-matured 'aquaporumab' anti-aquaporin-4 antibody for therapy of seropositive neuromyelitis optica spectrum disorders. Neuropharmacology..

[120] Davoudi V, Keyhanian K, Bove RM, Chitnis T (2016). Immunology of neuromyelitis optica during pregnancy. Neurol Neuroimmunol Neuroinflamm..

[121] Klawiter EC, Bove R, Elsone L, Alvarez E, Borisow N, Cortez M, Mateen F, Mealy MA, Sorum J, Mutch K, Tobyne SM, Ruprecht K, Buckle G, Levy M, Wingerchuk D, Paul F, Cross AH, Jacobs A, Chitnis T, Weinshenker B (2017). High risk of postpartum relapses in neuromyelitis optica spectrum disorder. Neurology..

[122] Collongues N, Alves Do Rego C, Bourre B, Biotti D, Marignier R, da Silva AM, Santos E, Maillart E, Papeix C, Palace J, Leite MIS, De Seze J. Pregnancy in patients with AQP4-Ab, MOG-Ab, or double-negative neuromyelitis optica disorder. Neurology. 2021;96(15):e2006-e2015. 10.1212/WNL.0000000000011744 33627499

[123] Borisow N, Hellwig K, Paul F (2018). Neuromyelitis optica spectrum disorders and pregnancy: relapse-preventive measures and personalized treatment strategies. EPMA J..

[124] Mao-Draayer Y, Thiel S, Mills EA, Chitnis T, Fabian M, Katz Sand I, Leite MI, Jarius S, Hellwig K (2020). Neuromyelitis optica spectrum disorders and pregnancy: therapeutic considerations. Nat Rev Neurol..

[125] Shosha E, Pittock SJ, Flanagan E, Weinshenker BG (2017). Neuromyelitis optica spectrum disorders and pregnancy: Interactions and management. Mult Scler..

[126] Chitnis T, Ness J, Krupp L (2016). Clinical features of neuromyelitis optica in children: US Network of Pediatric MS Centers report. Neurology..

[127] Tenembaum S, Yeh EA; Guthy-Jackson Foundation International Clinical Consortium (GJCF-ICC). Pediatric NMOSD: A Review and Position Statement on Approach to Work-Up and Diagnosis. Front Pediatr. 2020;8:339. 10.3389/fped.2020.00339 PMC7330096 32671002

[128] Juryńczyk M, Weinshenker B, Akman-Demir G (2016). Status of diagnostic approaches to AQP4-IgG seronegative NMO and NMO/MS overlap syndromes. J Neurol..

[129] Kim SH, Jang H, Park NY, Kim Y, Kim SY, Lee MY, Hyun JW, Kim HJ. Discontinuation of immunosuppressive therapy in patients with neuromyelitis optica spectrum disorder with aquaporin-4 antibodies. Neurol Neuroimmunol Neuroinflamm. 2021 Feb 23;8(2):e947. doi: 10.1212/NXI.0000000000000947. Erratum in: Neurol Neuroimmunol Neuroinflamm. 2021;8(4): PMID: 33622675; PMCID: PMC7903808. 10.1212/NXI.0000000000000947 PMC7903808 33622675

[130] Steinman L, Bar-Or A, Behne JM (2016). Restoring immune tolerance in neuromyelitis optica: Part I. Neurol Neuroimmunol Neuroinflamm..

